# Structural characterization of pyruvic oxime dioxygenase, a key enzyme in heterotrophic nitrification

**DOI:** 10.1128/jb.00342-24

**Published:** 2025-01-08

**Authors:** Shuhei Tsujino, Yusuke Yamada, Miki Senda, Akihiko Nakamura, Toshiya Senda, Taketomo Fujiwara

**Affiliations:** 1 Department of Microbiology and Immunology, Faculty of Medicine, Hokkaido University 12810 , Sapporo, Hokkaido, Japan; 2 Department of Environment and Energy Systems, Graduate School of Science and Technology, Shizuoka University 13058 , Shizuoka, Japan; 3 International Center for Synchrotron Radiation Innovation Smart (SRIS), Tohoku University 13101 , Sendai, Japan; 4 Structural Biology Research Center, Photon Factory, Institute of Materials Structure Science, High Energy Accelerator Research Organization 13551 , Tsukuba, Japan; 5 Department of Applied Life Sciences, Faculty of Agriculture, Graduate School of Science and Technology, Shizuoka University 13058 , Shizuoka, Japan; 6 Institute for Molecular Science, National Institutes of Natural Sciences 529437 , Okazaki, Aichi Prefecture, Japan; 7 Department of Materials Structure Science, School of High Energy Accelerator Science, The Graduate University of Advanced Studies, Tsukuba, Ibaraki, Japan; University of Florida Department of Microbiology and Cell Science, Gainesville, Florida, USA

**Keywords:** pyruvic oxime dioxygenase, class II aldolase, heterotrophic nitrification, *Alcaligenes faecalis*

## Abstract

Nitrification by heterotrophic microorganisms is an important part of the nitrogen cycle in the environment. The enzyme responsible for the core function of heterotrophic nitrification is pyruvic oxime dioxygenase (POD). POD is a non-heme, Fe(II)-dependent enzyme that catalyzes the dioxygenation of pyruvic oxime to produce pyruvate and nitrite. To analyze the catalytic mechanism of POD, the crystal structure of POD from *Alcaligenes faecalis* (AfPOD) was determined at 1.76 Å resolution. The enzyme is a homotetramer, and the subunit structure is homologous to those of class II aldolases, in particular, a zinc-dependent L-fuculose-1-phosphate aldolase. The active site of the subunit is located at the bottom of a cleft formed with an adjacent subunit. The iron ion at the active site is coordinated by three histidines and three water molecules in an octahedral geometry. The putative oxygen tunnel was connected between the active site and the central cavity of the tetramer. The N-terminal region of AfPOD, which is essential for catalytic activity, is disordered in the crystal. Structure prediction with AlphaFold2 combined with mutational experiments suggested that the disordered N-terminal region adopts an α-helix conformation and participates in the formation of the active site. The catalytic mechanism of the dioxygenase reaction by POD is discussed on the basis of the molecular docking model.

**IMPORTANCE:**

Our knowledge of nitrification has increased considerably in recent decades with the discovery of new nitrifying microorganisms and the characterization of their biochemical processes. Some heterotrophic bacteria and fungi are known to show nitrification activities, but the molecular mechanisms have been poorly understood. Here, we performed a structural characterization of pyruvic oxime dioxygenase (POD), a key enzyme in heterotrophic nitrification that produces nitrite from ammonia using pyruvic oxime as an intermediate. Structural and enzymatic analyses revealed that POD is a unique dioxygenase with features such as an aldolase backbone, an N-terminal α-helix, and an oxygen tunnel. Our results provide insights not only into the molecular mechanisms but also into the design of specific inhibitors of heterotrophic nitrification.

## INTRODUCTION

Nitrification, the catabolic oxidation of ammonia, is a microbiological process that is essential to the global nitrogen cycle. In addition, understanding the diversity and metabolic mechanisms of nitrifying microorganisms would lead to applications such as preventing nitrogen loss from fertilized cropland and ammonia removal technology for wastewater treatment (1, 2).

It has long been known that heterotrophic microorganisms, including various species of bacteria and fungi, perform dissimilatory ammonia oxidation to nitrite or nitrate (3), termed heterotrophic nitrification. Heterotrophic nitrification is thought to contribute significantly to the nitrogen cycle in the environment due to its species richness and abundance (4). Bacteria of the genera *Alcaligenes* and *Pseudomonas* are known to perform heterotrophic nitrification, which produces nitrite from ammonia using pyruvate oxime (PO) as an intermediate metabolite (5–7). Ono et al. (8) reported the presence of pyruvate oxime dioxygenase (POD), a dioxygenase that converts PO to nitrite, from *Alcaligenes faecalis*. However, the molecular and catalytic properties of POD have not been fully investigated since its discovery.

Therefore, we purified POD from *A. faecalis* (AfPOD) and showed that AfPOD is a metalloenzyme that requires Fe(II) ion for catalytic activity. AfPOD is a homotetramer, and its amino acid sequence is classified as a class II aldolase (PF00596) (9). We also confirmed that POD is involved in heterotrophic nitrifying activity (9, 10). Sequence homology searches using genome databases revealed *pod* genes in many types of bacteria in the phyla Proteobacteria, Actinobacteria, and fungi in the phylum Ascomycota (9, 11). While heterotrophic nitrifying activities have not been reported from many microbial species with *pod* genes, this result suggests that heterotrophic nitrifying microbes with the *pod* gene contribute more to the nitrogen cycle in the environment than previously thought.

Here, we report the first X-ray crystal structure of a novel dioxygenase, AfPOD, at 1.76 Å resolution. While the overall structure of AfPOD is similar to the class II aldolase with the zinc ion in the active site, AfPOD has the iron ion in the active site. Although the N-terminal 18 amino acids are critical for the enzyme activity of AfPOD, the N-terminal region was disordered in the crystal. Structure prediction using AlphaFold2 suggests that the disordered N-terminal region of AfPOD adopts an α-helix conformation and is involved in the formation of the active site. The catalytic mechanism of the dioxygenase reaction by POD was discussed based on the predicted substrate-binding structure obtained by molecular docking simulation.

## MATERIALS AND METHODS

### Preparation of purified samples of AfPOD, AfPODdN18, and BWPOD

AfPOD was overexpressed in the *Escherichia coli* cells and purified by two chromatographic steps: anion-exchange chromatography on DEAE-Toyopearl 650 M gel (Tosoh, Tokyo, Japan) and gel filtration chromatography on Sephacryl S-200 (Cytiva, Marlborough, MA, USA), as described previously (9). Sample purity was confirmed by SDS-PAGE (12). The construction of the overexpression system of the variant enzyme (AfPODdN18) lacking the N-terminal 18 residues and the purification of the recombinant were performed by a similar procedure as for AfPOD with some modifications as described in the supplemental material. The construction of the expression plasmid, overexpression, and purification of the recombinant protein of POD from *Bradyrhizobium* sp. WSM3983 (BWPOD), which was successfully crystallized in the early stage of the project, is also described in the supplemental material.

### Crystallization of AfPOD, AfPODdN18, and BWPOD

Crystallization conditions were initially screened using PEGIon/PEGIon2 (Hampton Research, Aliso Viejo, CA, USA), Crystal Screen Cryo and 2 Cryo (Hampton Research), Wizard Screens I and II (Rigaku, Tokyo, Japan), and PEGsII (Qiagen, Hilden, Germany) with a Protein Crystallization System 2 (PXS2) at the Structural Biology Research Center, High Energy Accelerator Research Organization, Japan (13). The obtained crystallization conditions were further optimized manually using the sitting-drop vapor diffusion method. However, since AfPOD is gradually deactivated even at 0°C due to iron dissociation from the active site (9), the AfPOD in the crystal was an inactive form. Therefore, reconstitution with Fe(II) and subsequent crystallization of AfPOD were performed in an anaerobic chamber with mixed gas (N_2_/H_2_, 96:4, vol/vol) and Pd catalyst to maintain anaerobic conditions (14). Reconstitution of AfPOD with Fe(II) was performed in the reservoir solution supplemented with 1 mM FeSO_4_. AfPOD crystals were obtained at 20°C in 36% (vol/vol) PEG400, 0.2 M calcium acetate, 0.1 M HEPES (pH 7.5) with 5.0 mg mL^−1^ of purified AfPOD by the sitting-drop vapor-diffusion method (Fig. S1a). In addition, crystallization of the variant enzyme (AfPODdN18) lacking the N-terminal 18 residues was performed under aerobic conditions with Mn(II) ions. AfPODdN18 crystals were obtained at 20°C in 20% (wt/vol) PEG 3350, 1 mM manganese chloride, 0.2 M sodium tartrate (pH 7.3) with 5.0 mg mL^−1^ of purified AfPODdN18 by the sitting-drop vapor-diffusion method (Fig. S1b). Both crystals were successfully obtained under each condition as shown in Fig. S1. The BWPOD crystals were obtained at 20°C in 1.56 M ammonium sulfate and 25% (vol/vol) glycerol with 10 mg mL^−1^ of purified BWPOD by the sitting-drop vapor-diffusion method as described in the supplemental material.

### Data collection and structure determination

Crystals of AfPOD and BWPOD were cryoprotected with the corresponding crystallization solution. Crystals of AfPODdN18 were cryoprotected with the crystallization solution containing 30% (vol/vol) glycerol. All crystals were frozen in liquid nitrogen. The X-ray diffraction data sets of BWPOD were collected at −178°C using a beam wavelength of 2.7 Å at BL-1A of Photon Factory (Tsukuba, Japan) and processed using X-ray detection software (15). Phase determination and initial model construction of BWPOD were performed using CRANK2 (16) by the native SAD method (17). The X-ray diffraction data sets of AfPOD and AfPODdN18 were collected at −178°C using a beam wavelength of 0.98 Å at Photon Factory’s BL-17A. The diffraction data sets were processed using AutoPROC (18) due to the anisotropy of the crystals. Molecular replacement was performed with MOLREP using the structure of BWPOD as a search model (19). Model building and crystallographic refinement were performed using COOT, REFMAC5, and BUSTER (20–22).

The metal species that bind to the active site were identified using anomalous difference Fourier maps calculated from diffraction data sets with X-rays on the shorter and longer wavelength sides of their absorption edges. All crystallographic figures were generated using PyMOL (Schrödinger Inc., New York, NY, USA).

### Site-directed mutagenesis of AfPOD

Site-directed mutagenesis of the AfPOD was performed by the technical application of PCR. A set of oligonucleotide primers, in which the corresponding original amino residue was replaced by the mutation, was amplified using the AfPOD expression vector with a C-terminal His_6_-tag as a template. After treatment with the restriction enzyme *Dpn*I to digest the template DNA, the PCR product was introduced into the *E. coli* BL21-CodonPlus(DE3). The PCR product was cyclized by homologous recombination between the 5′- and 3′-regions in the host cells, generating the strain for the overexpression of the variant AfPOD. After confirmation of the nucleotide sequence, the resulting strain was cultured aerobically in the 2× YT medium (30 mL). Expression was performed in the same manner as for the wild-type (WT) AfPOD. The variant AfPOD was purified by His-tag purification using His-Spin Protein Miniprep (Zymo Research, Irvine, CA, USA) according to the manufacturer’s protocol. The primers used for the PCR amplification are listed in Table S1.

### Homology analysis and structure prediction

Alignment analysis and conserved amino acid estimation were performed using Clustal Omega (23), MEGA X (24), and ConSurf server (25). Comparison with protein structures deposited in the PDB was performed using the DALI server (26). Structure prediction of the N-terminal part of AfPOD was performed using AlphaFold2 (27) as implemented by the ColabFold server (28).

### Enzymatic analysis

POD activity was determined by measuring the rate of nitrite production in the assay solution containing 20 mM Tris-HCl buffer (pH 8.0), 1.0 mM sodium ascorbate, 100 µM FeSO_4_, and 1.0 mM pyruvic oxime. The concentration of nitrite was measured spectrophotometrically using a diazo-coupling method (29). Each reaction was run in triplicate. GraphPad Prism 9 (GraphPad Prism Software Inc., San Diego, CA, USA) was used for statistical data analysis. Pyruvic oxime was synthesized according to Quastel et al. (30). Protein concentration was measured using a BCA protein assay kit (Pierce, Rockford, IL, USA) with bovine serum albumin as the standard. Spectroscopic analysis in the visible region was performed in a 1 cm light path cuvette using a UV-2600 spectrophotometer (Shimadzu, Kyoto, Japan). All chemicals used in the experiments were of the highest quality commercially available.

### Docking simulation

In the present study, co-crystallization or soaking experiments did not yield the structure of AfPOD bound to pyruvic oxime. We attempted to dock the substrate with the AfPOD structure that was generated by removing the binding water molecules on Fe ion from the crystal structure (8IL8) and adding hydrogens at pH 7 using PyMOL. The docking simulation was performed using Gnina (https://github.com/gnina/gnina), a convolutional neural network (CNN) molecular docking program, with an accurate line option (31). The EcFucA (4FUA) was aligned with AfPOD using the three catalytic histidine residues. Search regions were defined using the autobox_ligand option with the superimposed phosphoglycohydroxamic acid (PGH).

## RESULTS

### Conserved amino acid analysis of AfPOD

**Fig 1:**
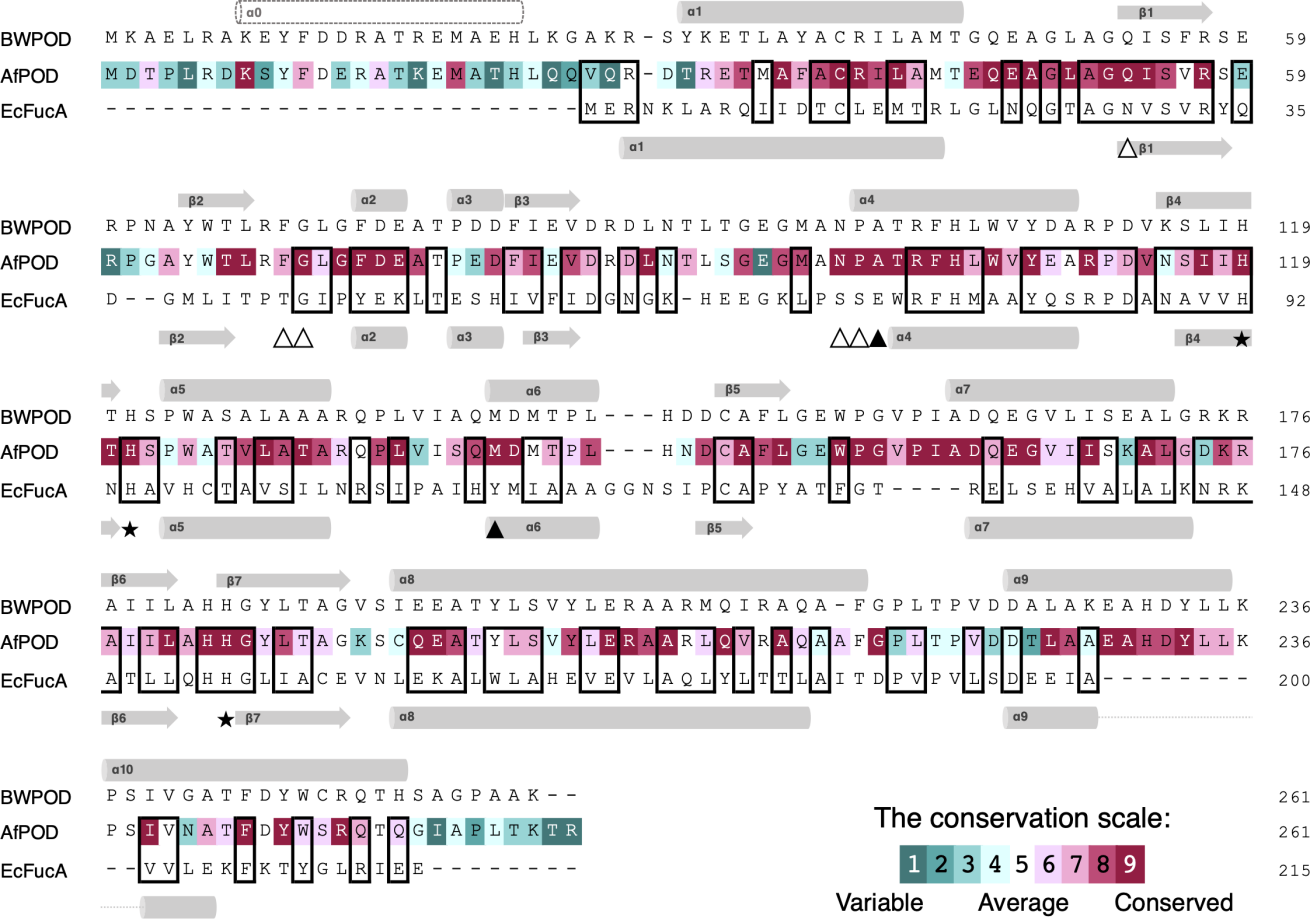
Sequence alignment of AfPOD, BWPOD, and EcFucA. The amino acid sequences of AfPOD, BWPOD, and EcFucA were aligned, and conserved amino acids are shown in boxes. Arrows and bars indicate α-helix and β-sheet structures, respectively. The amino acid sequence of AfPOD is indicated according to the conservation scale in the PODs. The amino acids of EcFucA for metal binding (His triad), substrate recognition, and catalytic residues are indicated by stars (★), white triangle (△), and black triangle (▲), respectively.

The internal region of 178 residues of AfPOD, excluding the N-terminal 32 residues and the C-terminal 51 residues, has been annotated as a class II aldolase (Pfam00596) and showed a 20.7% amino acid sequence identity with L-fuculose-1-phosphate aldolase (FucA), whose crystal structure has already been solved (9). Interestingly, a homology search using the KEGG genome database revealed that many class II aldolase genes with unknown functions shared extremely high sequence homology (*E*-value < e-60) with AfPOD. Molecular phylogenetic analysis has shown that these proteins form a monophyletic group that is distinct from other class II aldolases such as FucA, and recombinant experiments have confirmed their POD activity (11). The amino acid sequence alignment of AfPOD, BWPOD, and *E. coli* FucA (EcFucA) is shown in Fig. 1. The result of a conservation analysis of amino acid residues, which was conducted using the sequences of all 452 PODs identified in the KEGG genome database, is also shown in the figure. Since the His-triads (His119, His121, His183, and AfPOD numbering), corresponding to the zinc-binding motif of EcFucA, are completely conserved, they are predicted to coordinate the Fe(II) ion in the active site of the POD (9, 32). Highly conserved regions, such as Glu45-Ser55, Gly72-Ala76, Asn98-His104, and Trp155-Gly165 (AfPOD numbering), were found in the PODs. In addition, highly conserved amino acids such as Lys8 and Met19 were found in the N-terminal region, which is not observed in class II aldolases and is a unique feature of PODs.

### Overall structure of AfPOD

The crystal structures of AfPOD and AfPODdN18 were determined at resolutions of 1.76 and 1.55 Å, respectively (Table 1). Although crystal structure analysis of the AfPOD-substrate complex was attempted by the soaking method under anaerobic conditions, no electron density was observed for the substrate molecule.

**TABLE 1:** Data collection and refinement statistics

	AfPOD	AfPODdN18	BWPOD
Data collection			
Space group	*C*2	*P*2_1_2_1_2_1_	*I*432
Cell dimensions			
a, b, c (Å)	138.00, 107.55, 87.79	49.22, 135.05, 138.04	153.90, 153.90, 153.90
α, β, γ (°)	90, 128.07, 90	90, 90, 90	90, 90, 90
Resolution (Å)	76.4–1.76 (1.90–1.76)	96.5–1.55 (1.68–1.55)	76.9–2.47 (2.51–2.47)
*R*_pim_	0.035 (0.363)	0.042 (0.444)	0.014 (0.284)
I/σI	8.9 (1.5)	10.7 (1.7)	43.9 (2.6)
Completeness (%) spherical	55.6 (14.1)	71.1 (16.5)	100.0 (100.0)
Completeness (%) ellipsoidal	93.5 (74.2)	94.0 (61.2)	N/A^*a*^
Multiplicity	3.4 (3.2)	6.6 (5.2)	38.1 (39.4)
CC_1/2_	0.999 (0.706)	0.998 (0.630)	1.000 (0.868)
Refinement			
Resolution (Å)	76.43–1.76	22.68–1.55	76.95–2.47
No. reflections	55,281	95,105	11,502
*R*_work_/*R*_free_	0.174/0.207	0.180/0.204	0.227/0.257
No. atoms			
Protein	7,116	6,961	1,684
Ion	4	4	1
Water	693	682	21
B-factors			
Protein	32.14	19.50	61.94
Ion	26.52	15.73	55.47
Water	38.77	33.03	58.29
Root mean square deviations			
Bond lengths (Å)	0.0125	0.0138	0.0038
Bond angles (°)	1.53	1.50	0.93
Ramachandran plot			
Favored/allowed/outliers	98.03/1.54/0.44	97.12/2.32/0.55	97.24/2.30/0.46

^*a*^ N/A, not available.

**Fig 2:**
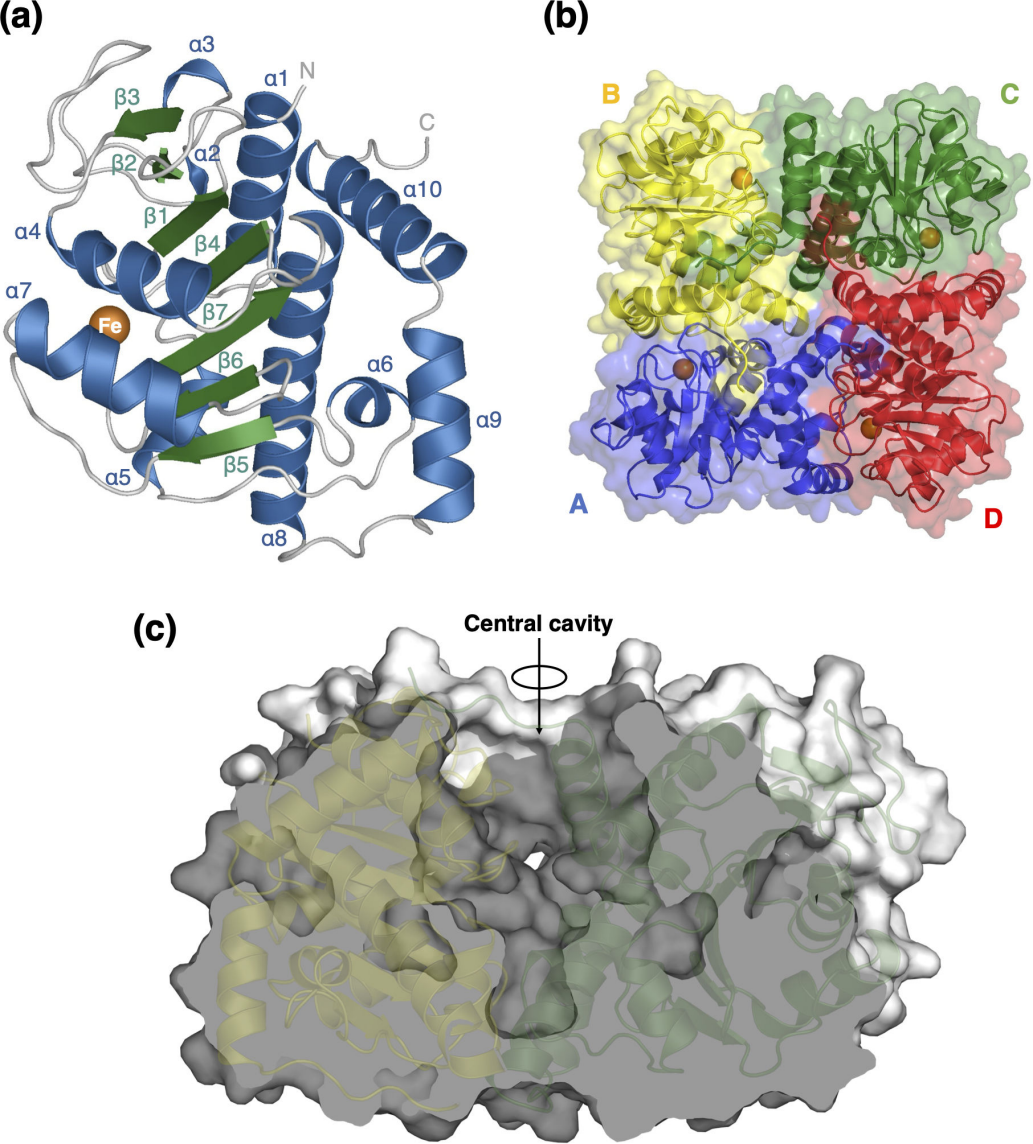
Monomeric and tetrameric structures of AfPOD. (**a**) Ten α-helices (blue) and seven β-sheets (green) in the monomeric structure of AfPOD. (**b**) Top view of the entire AfPOD molecule along the fourfold axis. Each subunit is indicated by a single color (A, blue; B, yellow; C, green; and D, red). (**c**) Cross-sectional view of the entire AfPOD molecule. The iron ions are shown in orange.

The final structure model of the AfPOD tetramer contains 920 amino acid residues, 693 water molecules, and 4 iron ions. The N-terminal region (Met1-Arg28) and the C-terminal region (Lys259-Arg261) of each subunit showed no apparent electron density. The AfPOD monomer consists of a seven-stranded anti-parallel β-sheet (β5↑, β6↑, β7↓, β4↑, β1↓, β2↑, and β3↓) flanked by two α-helices on one side (α4 and α7) and eight α-helices (α1, α2, α3, α5, α6, α8, α9, and α10) on the opposite side (Fig. 2a). AfPOD has a tetrameric structure with approximate dimensions of 60 × 40 × 80 Å^3^ (Fig. 2b). The central cavity of the tetramer has an 18 Å diameter opening on the C-terminal side and is closed by the last turn of α8 on the opposite side (Fig. 2c). The exposed surface area of the tetramer is approximately 45,000 Å^2^, of which 1,522 and 7 Å^2^ are buried at the interface between the A and B subunits and that between the A and C subunits, respectively. The solvation free energy (Δ*G*) gain calculated by PISA (33) is −19.2 kcal/mol between the A and B, the potential number of hydrogen bonds is 16, and the number of salt bridges is 10; while between the A and C, these values are −0.2 kcal/mol, 0, and 0, respectively.

### Putative active site structure

**Fig 3:**
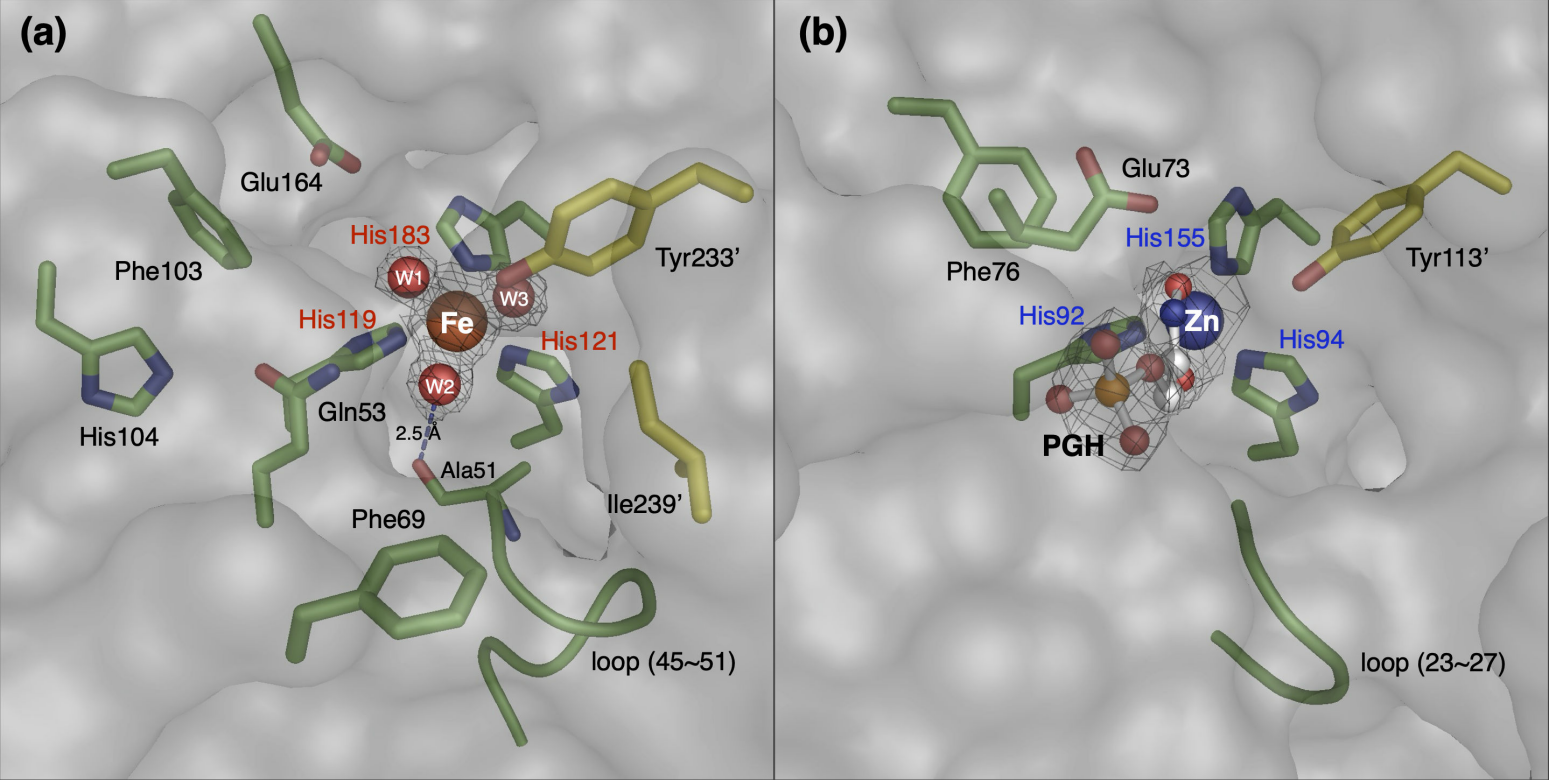
Comparison of the active site structures of AfPOD and EcFucA. The active site structure of AfPOD (8IL8) (**a**) was compared with that of EcFucA (4FUA) (**b**). The side chains and loop structure around the His triad are shown. Electron density maps around the iron and ligands are also shown. Residues shown in green are from subunit A, and the residues shown in yellow with an attached label (') are from the neighboring subunit B.

The anomalous difference Fourier maps revealed that the metal ion coordinated by three His residues was confirmed to be an iron ion, as expected from a previous biochemical analysis (Fig. S2a and b). In subunit A, the iron ion is located at a depth of about 8 Å at the bottom of an active site pocket with an opening of about 16 Å long and 8 Å wide at the boundary to the adjacent subunit B (Fig. 3a). The opening is constricted by the side chains of Phe69, Tyr233', and Ile239'. Here, an attached label (') to an amino acid indicates that it is derived from a neighboring subunit molecule. Imidazole nitrogen atoms of His119, His121, and His183 and oxygen atoms of three water molecules (W1, W2, and W3) coordinate the iron ion, forming an octahedral arrangement. The geometric parameters of the ligands are summarized in Table S2. The side chains of Gln53, Phe103, His104, and Glu164 were located around the iron center. These amino acid residues (His119, His121, His183, Gln53, Phe103, His104, and Glu164) are completely conserved in different PODs (Fig. 1). The α1-β1 loop (Glu45-Ala51), which is highly conserved among PODs, is also located near the iron center and is fixed by hydrogen bonding between the main-chain carbonyl oxygen of Ala51 and the iron-coordinated water 2 at distances of 2.5  Å. Interestingly, the side chain of Glu47 in the mobile loop shows a different conformation in subunits A, C and B, D (Fig. S3), which may affect the transport of oxygen molecules to the active site (see below).

### Mutagenesis analysis of AfPOD

Of the amino acids highly conserved between PODs, six amino acid residues located near the putative active site, Gln53, Phe103, His104, Glu164, Phe69, and Tyr233', were substituted by alanine, and the activity of each variant was assayed (Table 2). The catalytic constant (*k*_cat_) of the POD activity of the Q53A, H104A, E164A, and F69A variants was reduced to about 3%–5% of the value of the wild-type enzyme and was further reduced to 0.2%–0.3% in the F103A and Y233A variants. The Michaelis constant (*K*_*m*_) values for the Q53A, F103A, and H104A variants were approximately 0.1–0.2 mM in comparison to the wild-type enzyme (0.622 mM), indicating an enhancement of substrate affinity due to the mutations. All three variants, Q53A, F103A, and H104A, showed similar catalytic changes in increased substrate affinity, indicating that the Gln53, Phe103, and His104 residues are not only structurally but also functionally coordinated. In contrast, substitutions of Phe69 or Tyr233' to alanine significantly reduced substrate affinity. Tyr233' is conserved in all the PODs as an aromatic amino acid (Tyr, Trp, or Phe) (11). The side chains of Phe69 and Tyr233′ are 8.3 and 12.1 Å away from the iron center, respectively, and are not expected to interact directly with the substrate molecule bound to the iron center. The result suggests that the hydrophobic region formed by the side chains of Tyr233' and Phe69 in the vicinity of the active site is important for the enzymatic activity of POD.

**TABLE 2:** Kinetic parameters of wild-type and variant AfPODs^*a*^

	*k_cat_*/*K*_*m*_ (s^−1^ M^−1^)	*k*_cat_ (s^−1^)	*K*_*m*_ (mM)
WT	7.7 × 10^3^ (100)	4.797 ± 0.169 (100)	0.622 ± 0.083
dN18	N.D.	N.D.	N.D.
Q53A	1.7 × 10^3^ (22.0)	0.162 ± 0.008 (3.4)	0.096 ± 0.018
F103A	9.0 × 10 (1.2)	0.008 ± 0.001 (0.2)	0.089 ± 0.022
H104A	1.2 × 10^3^ (16.0)	0.210 ± 0.013 (4.4)	0.170 ± 0.037
E164A	2.9 × 10^2^ (3.7)	0.270 ± 0.020 (5.6)	0.943 ± 0.240
F69A	5.9 (0.1)	0.246 ± 0.018 (5.1)	41.509 ± 9.984
Y233A	0.3 (0.003)	0.012 ± 0.001 (0.3)	42.247 ± 16.705
K8E	9.2 (0.1)	0.632 ± 0.066 (13.2)	68.766 ± 20.299
E229K	2.1 × 10^3^ (27.6)	4.580 ± 0.098 (95.5)	2.150 ± 0.172
K8E/E229K	6.9 × 10^2^ (9.0)	1.762 ± 0.109 (36.7)	2.538 ± 0.568

^*a*^ The values in parentheses of *k*_cat_/*K*_*m*_ and *k*_cat_ indicate the relative value to WT. N.D., not detected.

### Structure prediction of the disordered N-terminal region

Since an AfPODdN18 variant lacking the N-terminal 18 amino acid residues showed no POD activity (Table 2), the N-terminal region should play an important role in the POD activity. However, this region was disordered in the AfPOD crystal; therefore, no structural information on the N-terminal region was obtained. The structure of AfPODdN18 crystallized in the coexistence of manganese was determined as a tetramer containing 918 amino acid residues, 682 water molecules, and 4 metal ions. The N-terminal regions (Met19-Arg28) and C-terminal regions (Lys259-Arg261) of each subunit showed no apparent electron densities. The overall structure of AfPODdN18 is nearly identical to the AfPOD with a Cα root mean square deviation (RMSD) of 0.3 Å (845 Cα atoms). The anomalous difference Fourier maps suggested that the electron density near the His triad in the AfPODdN18 is not a manganese ion (Fig. S2c and d). Nickel or cobalt ions may have been bound to the His triad, rather than manganese, during overexpression or purification.

**Fig 4:**
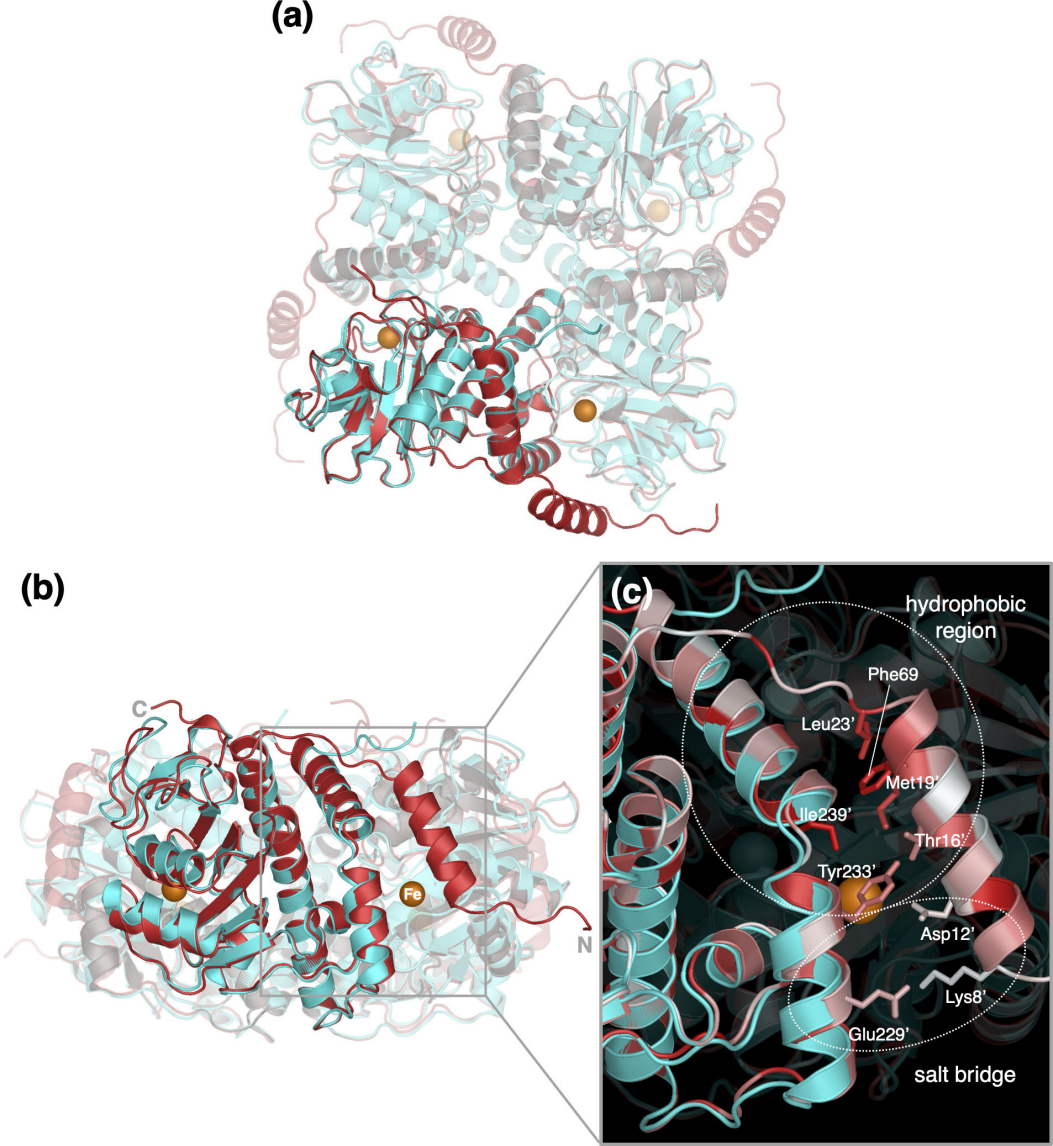
Comparison of the AfPOD crystal structure and the structure predicted by AlphaFold2. (**a**) Top and (**b**) side views of the structures. (**c**) The crystal structures (cyan) and their predicted structures (red) have been superimposed. The zoomed views show the active site of subunit A covered by an N-terminal α0-helix extending from the adjacent subunit B. The amino acid residues of subunit B are marked with an attached label. The hydrophobicity and hydrophilicity of the amino acid side chains are color coded in red and white, respectively.

AlphaFold2, a high accurate structure prediction software, has been reported to be useful for predicting the structure of disordered regions (34). Therefore, we used AlphaFold2 implemented on the ColabFold server to predict the structure of the entire length of the AfPOD, including the disordered region. The predicted structure showed a high agreement with the AfPOD crystal structure with a Cα RMSD of 0.5 Å (720 Cα atoms, Fig. S4). In the predicted structure, the N-terminus of the AfPOD forms an α-helix (Lys8-His22), and the C-terminus is further extended toward an adjacent subunit (Fig. 4). The N-terminal α-helix, which is designated as helix α0, of an adjacent subunit covers the putative active site pocket, and hydrophobic interactions between the side chains of Thr16', Met19', and Leu23' on the α-helix and those of Phe69, Tyr233', and Ile239' at the pocket opening were expected. It is reasonable to assume that helix α0 is a part of the active site.

The formation of a salt bridge by electrostatic interactions between the side chains of Lys8′ and Glu229′, which are highly conserved in POD, was also suggested (Fig. 1 and 4). Three variants, K8E, E229K, and K8E/E229K, were generated, and their POD activities were measured to assess the importance of the salt bridge (Table 2). The POD activity of the K8E variant was significantly reduced in both substrate affinity and catalytic activity and then the specificity constant (*k*_cat_/*K*_*m*_) was reduced to 0.1% of that of the WT enzyme. In the E229K variant, the catalytic constant was not affected, but the substrate affinity was reduced to about 30% of that of the WT enzyme. Interestingly, while the introduction of the K8E mutation into the wild-type enzyme resulted in a significant decrease in substrate affinity, the K8E mutation into the E229K variant had only a small effect on substrate affinity. This result can be interpreted as the substrate affinity and catalytic constant, which were significantly lowered in the K8E variant, were restored by the further introduction of the E229K mutation. Since Lys8 is the only basic amino acid near the active site, replacing it with an acidic amino acid could disrupt the structure of the active site by preventing salt bridge formation. It was suggested that the substitution of Glu229 in the K8E variant for basic Lys restores the structure of the active site by reforming the salt bridge between the two residues, resulting in some recovery of enzyme activity.

### Putative oxygen pathway

**Fig 5:**
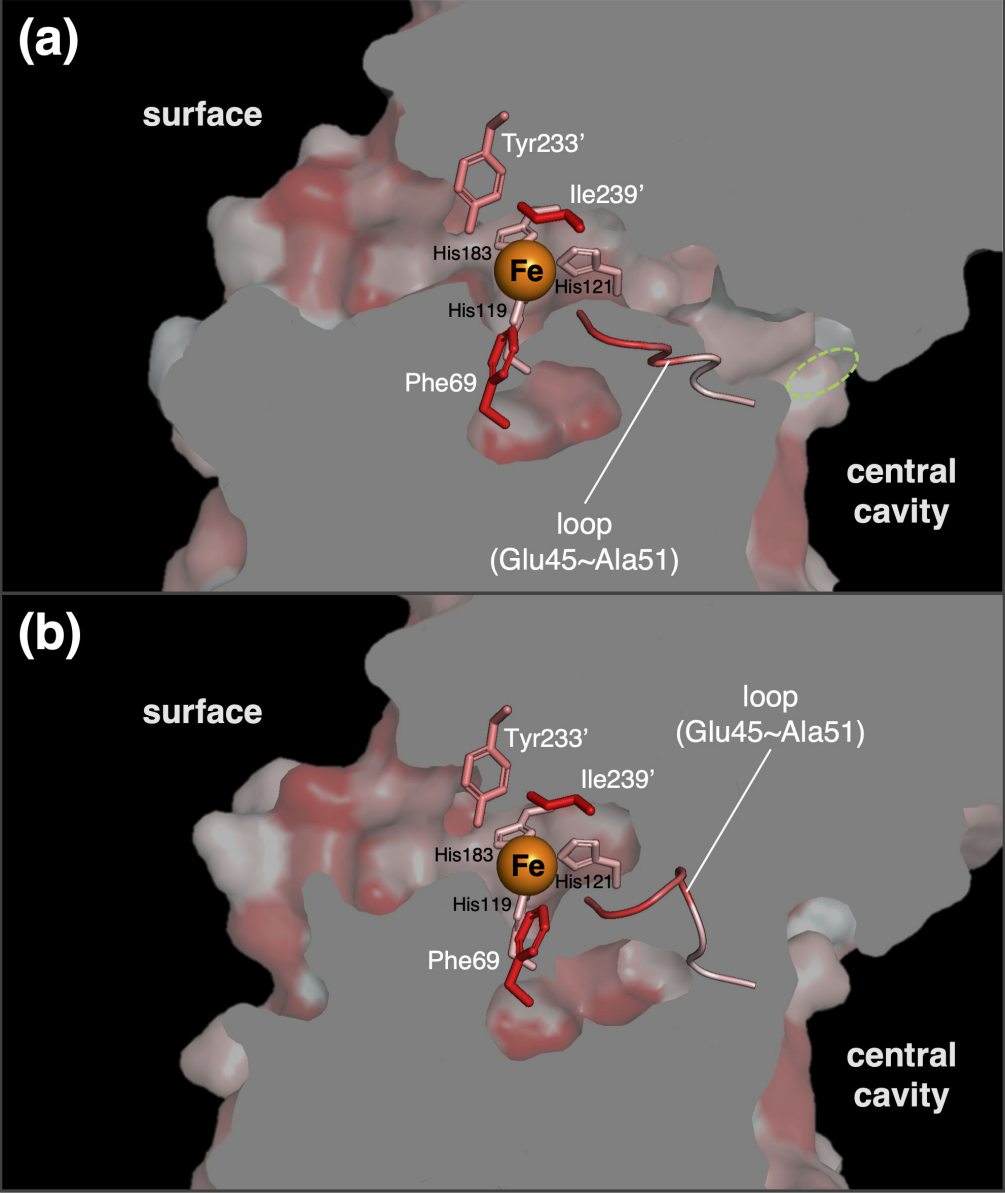
Cross-sectional view of the putative oxygen tunnel. The hydrophobicity of the tunnel interior leading to the active site of subunit A (**a**) and that subunit D (**b**) is color coded (red, hydrophobic; white, hydrophilic). The entrance of a tunnel leading to the active site is circled in green. His triad (His119, 121, and 183) and side chains around the active site pocket (Phe69, Tyr233', and Ile239') and loop structure (Glu45 –Ala51) are shown in the figure.

A putative oxygen pathway extending from the central cavity of the AfPOD tetramer to the active site has been identified. A 14 Å-long tunnel with a 3.5 Å diameter opening at the central cavity extends along the interface between the subunit molecules to the iron center (Fig. 5). Notably, the α1-β1 loop (Glu45-Ala51) forms part of the tunnel in subunits B and D. However, the tunnel is blocked by a conformational change of the Glu47 in subunits A and C, which probably prevents the passage of oxygen molecules (Fig. 5a and b). Since the deeper part of the tunnel and the active site pocket are hydrophobic, oxygen molecules may be recruited to the pocket by diffusion due to their hydrophobic nature (35). The structural difference of the α1-β1 loop suggested that the oxygen supply to the active site pocket of POD was controlled by the movement of the α1-β1 loop. The entrance to the active site pocket opened in the region proximal to the side chains of Phe69, Tyr233′, and Ile239′. In the closed state, where the N-terminal α-helix covers the pocket opening, this space is further covered by the hydrophobic side chains of Thr16′, Met19′ and Leu23′, making this space highly hydrophobic and suitable as an oxygen binding site.

### Docking simulation of the active site with pyruvic oxime

Since the crystal structure of AfPOD in the bound state with pyruvate oxime was not obtained in this experiment, we attempted to estimate the substrate binding structure of AfPOD by docking simulation. Docking with the Gnina program yielded a total of 20 structures with a CNN pose score greater than 0.4 (Table S3). In the three structures with the highest pose scores (models 1–3), the carbonyl oxygen and the oxime oxygen of the pyruvate oxime were each located at the positions of two of the three coordinated water molecules of iron. In the most likely model 1 structure, the carbonyl and oxime oxygens were located at positions W1 and W2, respectively, and the carbonyl carbon, C2-carbon, oxime nitrogen, and oxime oxygen of the pyruvate oxime formed a six-membered ring structure with Fe(II) (Fig. S5). The oxime oxygen of the pyruvic oxime was positioned at a distance of approximately 3 Å, allowing hydrogen bonding with the carbonyl oxygen of the Ala51 main chain and the amide nitrogen of the Gln53 side chain. The carboxyl oxygen of the substrate molecule was also predicted to form hydrogen bonds with the amide nitrogen of the Gln53 side chain and the carbonyl oxygen of the Glu164 side chain. The conserved amino acid residues Phe103, His104, Phe69, and Tyr233, which have been shown to be essential or strongly involved in POD activity, did not interact directly with the substrate molecule in the docking model.

## DISCUSSION

**Fig 6:**
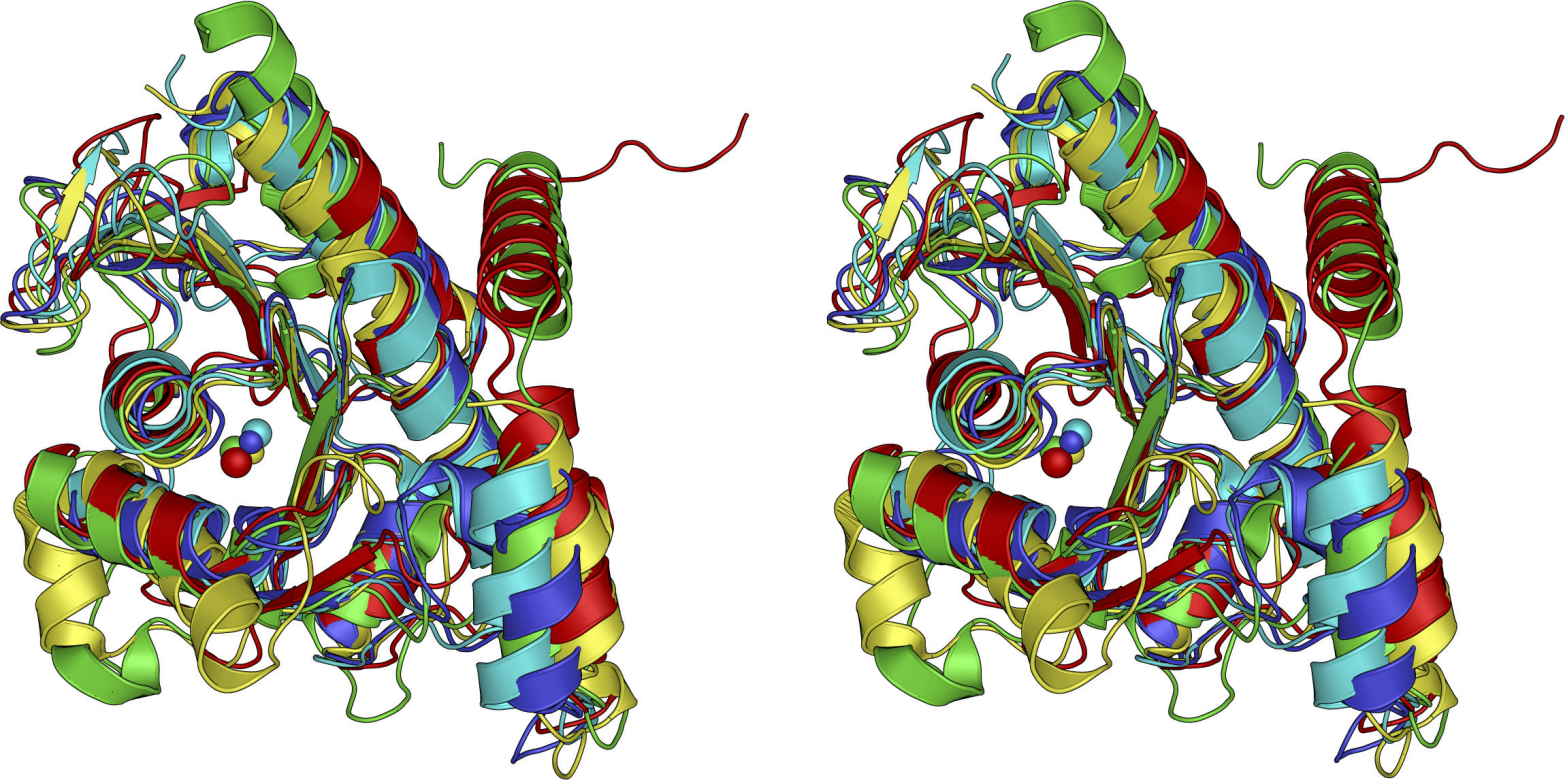
Stereoview diagram of the superposition of AfPOD with the homologous enzymes. The monomeric structures of the protein groups with particularly high similarity (*Z*-score ≥ 20.0) are superimposed based on the structural homology search on the DALI server. AfPOD (PDB code: 8IL8), EcFucA (1FUA), HMPDdc (2Z7B), DrdA (6BTG), and EcAraD (1JDI) are shown in red, blue, green, cyan, and yellow, respectively.

While several cytochrome P450-type enzymes are known to catalyze the monooxygenation of oxime (aldoxime) compounds to form nitriles (36), POD is the only dioxygenase that uses oxime compounds as substrates to produce nitrite. As expected from the results of the sequence homology analysis, AfPOD showed remarkable structural homology to class II aldolases, which are zinc-dependent lyases. A structural similarity search using the DALI server revealed that AfPOD is structurally similar to class II aldolases (COG0235) with known functions, such as FucA, 5-deoxyribose disposal aldolase (DrdA), L-ribulose-5-phosphate 4-epimerase (AraD), and L-rhamnulose-1-phosphate aldolase (Rua). FucA showed the highest structural homology to AfPOD among them. These enzymes require a zinc ion for their activity. In addition, AfPOD showed a structural similarity to Mn(II)-dependent 3-hydroxy-2-methylpyridine-4,5-dicarboxylate decarboxylase (HMPDdc) (37–40) (Table S4). No dioxygenases showed structural similarity to AfPOD. Superposition of the AfPOD subunit with EcFucA (PDB code: 1FUA), HMPDdc (2Z7B), DrdA (6BTG), and EcAraD (1JDI) (Fig. 6) showed that the seven-stranded, anti-parallel β-sheet structure flanked by α-helices from both sides of the β-sheet is well conserved among them. PODs have additional sequences at the N-terminus consisting of 20–30 amino acid residues that are not present in class II aldolases, corresponding to the disordered region of AfPOD in its crystal structure.

The active site of AfPOD has an iron ion that has an octahedral coordination sphere with six ligands: three His residues and three water molecules (Table S2). On the other hand, with a few exceptions such as cysteine dioxygenase, the non-heme Fe(II)-dependent dioxygenases have an iron center with two His residues and one Glu (or Asp) residue (41, 42). One possible explanation is that POD is the enzyme that was evolved by metal substitution using the protein folding common to the class II aldolases. The scenario that an oxidoreductase (dioxygenase) evolved from a lyase (aldolase) by replacing the zinc ion with the iron ion is unique in terms of the molecular evolution of metalloenzymes and should be further investigated.

The reaction mechanism of FucA has already been analyzed, and important catalytic residues have been identified. In Fig. 3, the structure of the active site of the AfPOD was compared with that of the EcFucA bound to the inhibitor PGH (PDB code: 4FUA). The aldol cleavage of L-fuculose-1-phosphate by FucA is initiated by the coordination of the carbonyl oxygen at the C2 position and the hydroxyl oxygen at the C3 position of L-fuculose in the open chain state to the zinc ion to stabilize the cis-enediolate structure. Cleavage of the C3-C4 bond is then promoted by deprotonation of the C4 hydroxyl group by the Tyr113' and that of the C3 hydrogen by the Glu73 (43). Two amino acids of EcFucA, Glu73 and Tyr113', which are essential for the catalytic function of FucA, are not conserved in the AfPOD sequence (Fig. 1) (44). The amino acid residues, Asn29, Thr43, Gly44, Ser71, and Ser72, which are involved in substrate recognition of EcFucA by hydrogen bonding to the phosphate group of PGH, were also not conserved in the active site pocket of AfPOD. However, Glu164 and Tyr233' in the active site of the AfPOD are structurally conserved with EcFucA; Glu164 and Tyr233' are located at the positions corresponding to Glu73 and Tyr113' of EcFucA, respectively (Fig. 3). Phe76 in EcFucA, which was proposed to interact with protonated Glu73 side chains, was conserved as Phe103 in AfPOD (Fig. 1 and 3) (32). The α1-β1 loop (Asn23-Ala27) of EcFucA, which corresponds to the α1-β1 loop (Glu47-Ala51) of AfPOD, is also highly mobile and has been suggested to be essential for substrate binding by the point mutagenesis experiment conducted on EcFucA (45). The main chain carbonyl oxygen of Ala27 has been proposed to be involved in the rearrangement of the hydrogen bond network upon substrate binding, leading to an induced fit at the active site of EcFucA (43). In the case of AfPOD, the movement of the α1-β1 loop may be involved in the regulation of O_2_ supply to the active site by switching the oxygen tunnel open/closed (Fig. 5). In addition, the main chain carbonyl oxygen of the corresponding Ala51 of AfPOD is thought to promote the POD reaction by hydrogen bonding with the substrate molecule, as discussed below.

**Fig 7:**
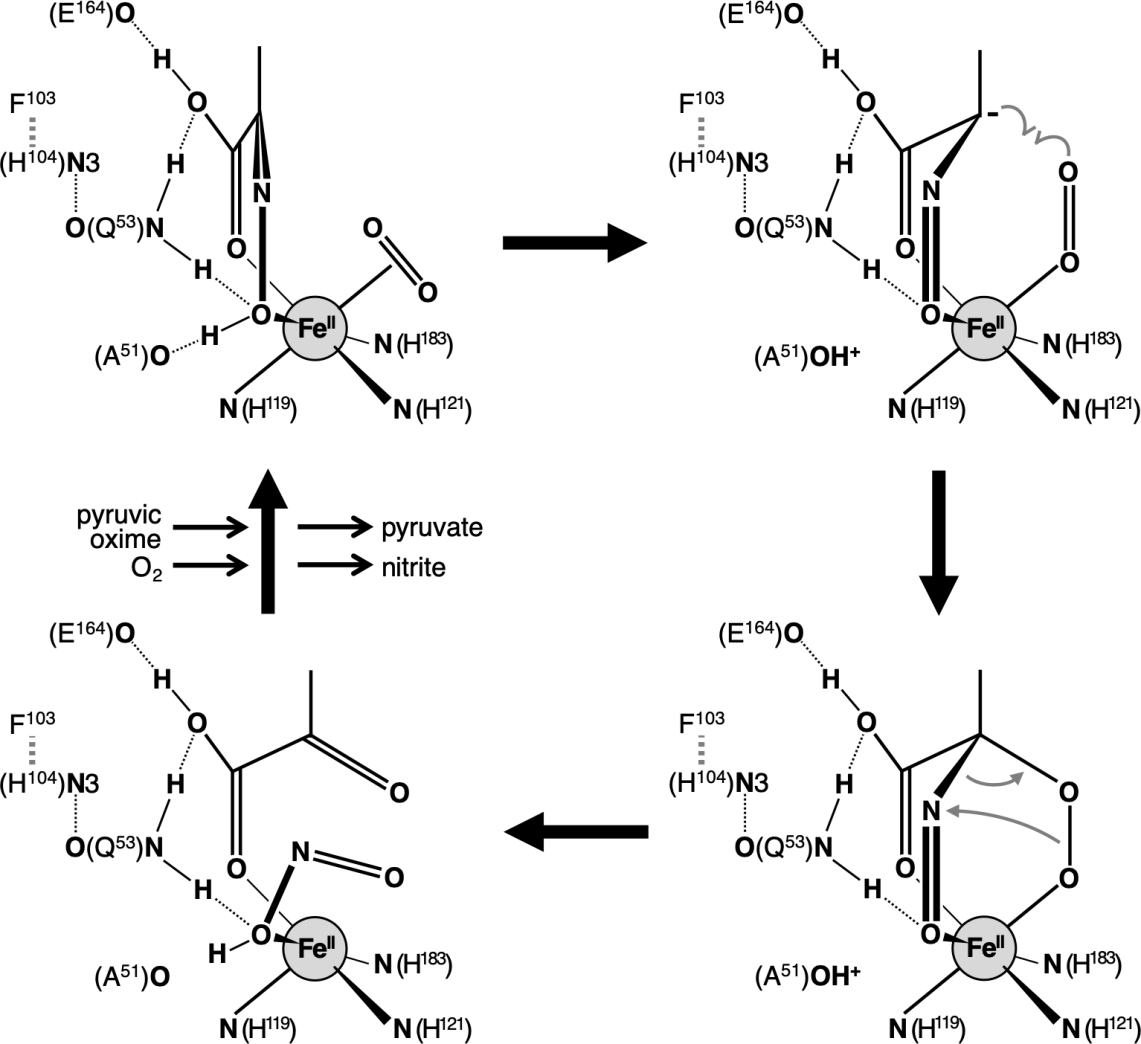
Proposed reaction mechanism of POD. Dotted line and thick dotted line show hydrogen bonding and π-π stacking interactions, respectively. See text for further details.

Based on the docking model 1 with the highest CNN pose score and the known catalytic mechanism of extradiol-type dioxygenase, the molecular mechanism of catalysis by POD was predicted as shown in Fig. 7. In the model 1 structure, the carbonyl and oxime oxygens of the pyruvic oxime are located at positions W1 and W2, respectively, forming a six-membered ring containing Fe(II) (Fig. S5). In addition, the oxime oxygen of the substrate molecule is hydrogen bonded to the carbonyl oxygen of the Ala51 main chain and the amide nitrogen of the Gln53 side chain, and the carboxyl oxygen is hydrogen bonded to the amide nitrogen of the Gln53 side chain and the carbonyl oxygen of the Glu164 side chain, respectively. Aldol reactions catalyzed by class II aldolases proceed via the tautomeric enol form of the substrate molecule, which is stabilized by the interaction of the carbonyl oxygen with an electron-withdrawing zinc ion (43). In the pyruvic oxime molecule, the oxime group (=N-OH) is in tautomeric equilibrium with the nitroso group (-N=O), and the equilibrium is usually strongly biased toward the oxime-type structure. Within the six-membered ring in docking model 1, the equilibrium is predicted to shift toward the nitroso form due to the electron-withdrawing effect of Fe(II) as a Lewis acid. In addition, the carbonyl oxygen of the main chain of Ala51 is close enough to form a hydrogen bond with the oxime oxygen and may contribute to the stabilization of the nitroso-form structure by accepting the oxime hydrogen. The shift in the oxime/nitroso tautomeric equilibrium and the deprotonation would lead the C2 carbon of the pyruvic oxime to an electron-rich state. O_2_ molecules entering the active site through the oxygen channel are expected to bind to the W3 position near the channel opening (Fig. 3a). A new carbon-oxygen bond is formed between the O_2_ and the C2 carbon, forming a six-membered transition state ring consisting of the C2 carbon, the nitroso nitrogen, the nitroso oxygen, two oxygen atoms, and an iron atom. Subsequent isomerization of the six-membered ring transition state, i.e., cleavage of the oxygen-oxygen bond and attack of the nitrogen by the oxygen atom on the iron, would result in the formation of nitrite and pyruvate.

In the structure of the docking model 1, hydrogen bonds with the side chains of Gln53 and Glu164 and with the main chain of Ala51 are expected to contribute to the binding of the pyruvic oxime molecule to the active site of AfPOD. A significant decrease in the catalytic constant *k*_cat_ was observed in the Q53A and E164A variants compared to the wild-type enzyme. On the other hand, the substrate affinity increased in the Q53A variant, whereas it was little affected in the E164A variant (Table 2). Interestingly, the F103A and H104A variants also exhibited decreased catalytic activity and increased substrate affinity observed in the Q53A variant. The side chains of the three amino acid residues Gln53, Phe103, and His104 are located close to each other within the putative active site, and π-π stacking interactions between the aromatic rings of Phe103 and His104, and hydrogen bonds between the imidazole N3 nitrogen of His104 and the carbonyl oxygen of the Gln53 side chain were indicated by crystal structure analysis (Fig. S5). In the presumed reaction mechanism shown in Fig. 7, the C2 carbon of the pyruvate oxime and the O_2_ molecule bonded to the W3 position would need to face each other in an appropriate configuration. These results suggest that the Phe103 and His104 side chains may indirectly promote the catalytic reaction by anchoring the Gln53 side chain in the appropriate position in the active site. In the Q53A mutant, the carbamoylethyl side chain is replaced by a small methyl side chain, which would facilitate the entry of the substrate molecule into the active site. However, it will be less likely to hold the substrate molecule in the appropriate spatial arrangement within the active site. The enzymatic properties observed in the F103A and H104A variants, which are similar to those of the Q53A variant, can be attributed to the inability to anchor Gln53 in its proper position.

The dN18 variant of AfPOD completely lost its enzymatic activity, indicating that the POD-specific N-terminal extension of about 30 amino acid residues, which is not present in other class II aldolases, is essential for catalytic activity (Table 2). Initially, we expected the flexibility of the region to cause disorder in the N-terminal sequence of AfPOD and BWPOD crystals, but the AlphaFold2 prediction gave an α-helix conformation in the sequence (Fig. 4). The active site pocket of subunit A was predicted to be covered by this N-terminal helix, designated α0, extending from subunit B. However, in the crystal, it could not assume such a configuration due to steric hindrance with other molecules in the lattice, and thus appeared to be observed as in the disordered state. Electrostatic interaction to form a salt bridge between the side chains of Lys8′ on the α0 helix and the Glu229′ on the α9 helix was also postulated to be involved in the “closed” state in which the α0 helix covers the active site pocket (Table 2; Fig. 4). No entrance of the substrate to the active site was found when the α0 helix was in the closed state. Therefore, the α0 helix would have to switch to the “open” state for the substrate molecule to enter the active site. At present, it is most acceptable to assume that this conformational switching between the closed and the open state of the α0 helix plays an important role in the uptake of the substrate. When the α0 helix returns to the closed state after substrate uptake, a hydrophobic environment is expected to be formed in the active site pocket as shown in Fig. 4. This hydrophobic nature may contribute not only to the recruitment of O_2_ molecules by diffusion through the tunnel but also to the formation of a suitable environment for the dioxygenase reaction in the active site pocket. The marked decrease in substrate affinity observed in the F69A, Y233A, and K8E mutants may be due to some impairment in the formation of the hydrophobic environment in the active site (Table 2).

In this study, the crystal structure of POD from the heterotrophic nitrifying bacterium *A. faecalis* was determined. The structure of AfPOD and its homologous enzyme BWPOD were very similar to those of class II aldolases, whereas the zinc ion, which is the active center in other class II aldolases, was replaced by Fe(II) ion in the active state of AfPOD. Class II aldolases catalyze aldol reactions by shifting the keto-enol tautomeric equilibrium of the substrate molecule to the enol side through the electron-withdrawing effect of the zinc ion. In POD, the electron-withdrawing action of Fe(II) ions is thought to shift the oxime-nitroso tautomeric equilibrium of the substrate molecule to the nitroso side, thereby promoting the reaction with Fe(II)-bound O_2_ and the removal of the nitroso group. An extended sequence at the N-terminus of POD, not present in other class II aldolases, was disordered in the crystal structure of AfPOD but was inferred to form an α-helix and positioned to cover the active pocket by the AlphaFold2 structure prediction. The conformational switching of this α0 helix between the open and closed states is thought to be important for the catalytic process of POD. The catalytic mechanism discussed above should be verified based on the crystal structure of substrate-binding state and by theoretical calculations. For example, the AfPOD variants with increased substrate affinity might be useful for preparing crystals of the substrate-binding enzyme. Furthermore, the conformational switching of the α0 helix of AfPOD could be directly confirmed by cryo-EM observations.

### Conclusions

AfPOD is a unique dioxygenase with high amino acid sequence similarity to class II aldolases such as EcFucA. We have determined the crystal structure of POD at 1.76 Å resolution, revealing a class II aldolase-like overall structure. The active site of AfPOD contains the Fe(II) that is coordinated by three His residues and three water molecules. While functionally important N-terminal residues were not observed in the crystal structure, a combination of the AlphaFold2 structure prediction and point mutation experiments suggested the critical function of the N-terminal (putative) α-helix in the catalytic reaction. In addition, the functional roles of amino acid residues around the iron center and the migration pathway of oxygen molecules were inferred. The catalytic mechanism of the POD was discussed on the basis of the molecular docking with the pyruvic oxime. This finding raises the possibility that class II aldolases of unknown function, present in the genomes of many microorganisms, may function as dioxygenases and be responsible for diverse metabolic functions other than aldolases. The findings also provide a platform for the future design of nitrification inhibitors specific for heterotrophic nitrification.

## Data Availability

The coordinates and the structure factor amplitudes for the structure of AfPOD, AfPODdN18, and BWPOD have been deposited in the Protein Data Bank (PDB) under accession codes 8IL8, 8IQA, and 8IX6.

## SUPPLEMENTAL MATERIAL

Supplemental figures and tables jb.00342-24-s0001.docx Fig. S1 to S5; Tables S1 to S4.

ASM does not own the copyrights to Supplemental Material that may be linked to, or accessed through, an article. The authors have granted ASM a non-exclusive, world-wide license to publish the Supplemental Material files. Please contact the corresponding author directly for reuse.
