# Supplementary material for: Structural characterization of pyruvic oxime dioxygenase, a key enzyme in heterotrophic nitrification
Source: J Bacteriol. 2025 Jan 8;207(2):e00342-24. doi: 10.1128/jb.00342-24 (PMC11841055; doi:10.1128/jb.00342-24)
Supplement: Supplemental figures and tables — Fig. S1 to S5; Tables S1 to S4. [file jb.00342-24-s0001.docx]

*Supplemental materials*

**Structural characterization of pyruvic oxime dioxygenase, a key enzyme of heterotrophic nitrification.**

Shuhei Tsujino^1,2#^, Yusuke Yamada^3^, Miki Senda^4^, Akihiko Nakamura^5,6^, Toshiya Senda^4,7^

and Taketomo Fujiwara^2#^

^1^Department of Microbiology and Immunology, Faculty of Medicine, Hokkaido University, Kita15 Nishi7, Kita-ku, Sapporo, Hokkaido 060-8638, JAPAN

^2^Department of Environment and Energy Systems, Graduate School of Science and Technology,

Shizuoka University, 836 Oh-ya, Suruga-ku, Shizuoka 422-8529, JAPAN

^3^International Center for Synchrotron Radiation Innovation Smart (SRIS), Tohoku University, 468-1 Aramaki-Aza-Aoba, Aoba-ku, Sendai, 980-8572, JAPAN

^4^Structural Biology Research Center, Photon Factory, Institute of Materials Structure Science,

High Energy Accelerator Research Organization, 1-1 Oho, Tsukuba 305-0801, JAPAN

^5^Department of Applied Life Sciences, Faculty of Agriculture, Graduate School of Science and Technology, Shizuoka University, 836 Oh-ya, Suruga-ku, Shizuoka 422-8529, JAPAN

^6^Institute for Molecular Science, National Institutes of Natural Sciences, 5-1 Higashiyama Myodaijicho, Okazaki, Aichi 444-8787, JAPAN

^7^Department of Materials Structure Science, School of High Energy Accelerator Science, The Graduate

University of Advanced Studies (Soken-dai), 1-1 Oho, Tsukuba, Ibaraki 305-0801, JAPAN

**Supplementary Methods**

***Construction of the AfPODdN18 expression system***

A set of oligonucleotide primers, AfpoddN18f and AfpodR, was designed for the construction of the mutant AfPOD lacking the 18 N-terminal amino acid residues (AfPODdN18) expression vector. Amplification was performed using KOD-plus DNA polymerase (Toyobo, Osaka, Japan) and the AfPOD expression vector (pAfPOD) as a template (**1**). The resulting PCR product was cloned into a pCR-blunt TOPO II vector (Invitrogen, Carlsbad, CA), yielding pCRAfPODdN18. After confirmation of the nucleotide sequence, the insert of pCRAfPODdN18 was digested with both *Nde*I and *Xho*I, and then cloned into the same restriction site of a pET21a+ vector (Novagen, Darmstadt, Germany), yielding the expression plasmid pAfPODdN18. The pAfPODdN18 plasmid was introduced into *E. coli* BL21-CodonPlus(DE3) (Agilent Technologies, Santa Clara, CA) to generatie the strain AfPdN18 for overexpression of AfPODdN18. Standard protocols used for DNA handling in *E. coli* were followed Sambrook and Russell (**2**). Primers used for the amplification are listed in **Supplementary Table S1**.

***Purification of AfPODdN18***

The purification of AfPODdN18 was performed as described above with slight modification using AKTA FPLC (Cytiva). The strain AfPdN18 was cultured aerobically in the 2×YT medium (1 L) supplemented with 50 μg mL-1 ampicillin at 37°C with reciprocal shaking at 150 rpm. At a mid-exponential growth stage (OD600 = 0.6–0.8), isopropyl β-D-1-thiogalactopyranoside (IPTG) was added to the medium to reach 0.3 mM for induction of AfPODdN18. After incubation at 20°C with shaking at 150 rpm for 3 h, the cells were collected by centrifugation. Cultured AfPdN18 cells were suspended in 30 mL of 20 mM Tris-HCl (pH 8.0) containing 10 μM phenylmethylsulfonyl fluoride (PMSF) (buffer A) and disrupted by sonication. After removal of unbroken cells by centrifugation at 12,000 × g for 10 min, the supernatant obtained was centrifuged at 140,000 × g for 65 min. The soluble fraction thus obtained was applied to a HiTrap DEAE FF anion-exchange chromatography column (Cytiva) equilibrated with buffer A. The recombinant protein adsorbed on the column was eluted by a linear gradient generated from 100 mL each of buffer A and buffer A containing 0.4 M NaCl. The fractions confirmed by SDS-PAGE were collected and then concentrated by 30–50% saturated ammonium sulfate fractionation. The precipitate obtained was suspended in 500 μL of buffer A containing 0.25 M NaCl and then applied to a gel filtration chromatography column of Superdex 200 Increase 10/300 GL (Cytiva) equilibrated with the same buffer. The fractions confirmed by SDS-PAGE were collected and concentrated by ammonium sulfate fractionation as above. The resulting precipitate was suspended in 10 mM HEPES buffer (pH 7.0) and dialyzed three times against the same buffer. The purified protein was electrophoretically homogenized by SDS-PAGE and then used for crystallization. All purification experiments were carried out at low temperatures around 4ºC.

***Construction of the BWPOD expression system and purification of the recombinant BWEPOD***

The gene encoding BWPOD, whose nucleotide sequence was optimized according to an *E. coli* codon usage, was synthesized (GENEWIZ Inc, South Plainfield, NJ). The 777 bp fragment was cloned into an *Nde*I and *Xho*I site of a pET21a(+) vector, yielding the expression plasmid pBWPOD. The pBWPOD plasmid was introduced into *E. coli* BL21-CodonPlus(DE3) to generate strain BW01 for overexpression of BWPOD. The His_6_-tagged BWPOD was overexpressed in strain BW01 and purified using a Ni^2+^-chelating Sepharose column (Cytiva).

**REFERENCES**

1. Tsujino S, Uematsu C, Dohra H, Fujiwara T. 2017. Pyruvic oxime dioxygenase from heterotrophic nitrifier *Alcaligenes faecalis* is a nonheme Fe(II)-dependent enzyme homologous to class II aldolase. *Sci Rep* 7:39991.
2. Sambrook J, Russell DW. 2001. Molecular cloning: a laboratory manual, 3rd edn. Cold Spring Harbor Laboratory Press, New York.

**Supplementary Table S1. Primers used in this study.**

| **Name** | **Nucleotide sequence (5' to 3')** | **Remarks (underlined)** |
| --- | --- | --- |
| AfpoddN18f | AGA TAT ACA TAT GGC CAC TCA TCT GCA ACA | *Nde*I recognition site was introduced into the inert ATG encoding M19 to become the transcription start codon. |
| AfpodR | GCT CGA GTC AGC GAG TTT TAG TTA AGG GCG | *Xho*I recognition site was introduced into the termination codon. |
| AfPODQ53Af | TCT GGC GGG CGC GAT CAG CGT CCG TTC C | ^157^C/^158^A were replaced by G/C for the Q53A substitution. |
| AfPODQ53Ar | GGA CGC TGA TCG CGC CCG CCA GAC CGG C |  |
| AfPODF69Af | TAC TGG ACC CTG CGC GCT GGC CT | ^205^T/^206^T were replaced by G/C for the F69A substitution. |
| AfPODF69Ar | AGG CCA GCG CGC AGG GTC CAG TA |  |
| AfPODF103Af | CCA CCC GCG CTC ACC TGT GGG TCT ACG A | ^307^T/^308^T were replaced by G/C for the F103A substitution. |
| AfPODF103Ar | ACA GGT GAG CGC GGG TGG CCG GGT TGG |  |
| AfPODH104Af | ACC CGC TTT GCC CTG TGG GTC TAC GAG G | ^310^C/^311^A were replaced by G/C for the H104A substitution. |
| AfPODH104Ar | ACC CAC AGG GCA AAG CGG GTG GCC GGG T |  |
| AfPODE164Af | ATC GCC GAT CAG GCA GGC GTC ATC ATT TCC | ^491^A/^492^G were replaced by C/A for the E164A substitution. |
| AfPODE164Ar | AAT GAT GAC GCC TGC CTG ATC GGC GAT GG |  |
| AfPODY233Af | GCG CAC GAC GCT CTG CTC AAG CCC TCC AT | ^697^T/^698^A were replaced by G/C for the Y233A substitution. |
| AfPODY233Ar | CTT GAG CAG AGC GTC GTG CGC CTC GGC CG |  |
| AfPODK8Ef | CCA CTC AGA GAC GAA TCC TA | ^22^A was replaced by G for the K8E substitution. |
| AfPODK8Er | TAG GAT TCG TCT CTG AGT GG |  |
| AfPODE229Kf | ATA CGC TGG CGG CCA AGG CGC ACG A | ^685^G/^687^A were replaced by A/G for the E229K substitution. |
| AfPODE229Kr | TCG TGC GCC TTG GCC GCC AGC GTA T |  |

**Supplementary Table S2. Geometry of the iron coordination sphere in the active site of AfPOD.**

| **Subunit** | **A** | **B** | **C** | **D** | **Average** |
| --- | --- | --- | --- | --- | --- |
| **Bond distances (Å)** | | | | | |
| Fe-His119 Nε | 2.21 | 2.25 | 2.17 | 2.21 | 2.21 |
| Fe-His121 Nε | 2.23 | 2.17 | 2.24 | 2.21 | 2.21 |
| Fe-His183 Nε | 2.22 | 2.13 | 2.24 | 2.18 | 2.19 |
| Fe-Water 1 | 2.14 | 2.22 | 2.25 | 2.26 | 2.22 |
| Fe-Water 2 | 2.42 | 2.39 | 2.45 | 2.36 | 2.41 |
| Fe-Water 3 | 2.18 | 2.18 | 2.25 | 2.29 | 2.23 |
| **Bond angles (°)** | | | | | |
| His119 Nε-Fe-His183 Nε | 88.36 | 90.40 | 89.41 | 89.63 | 89.45 |
| His121 Nε-Fe-His119 Nε | 97.81 | 98.47 | 98.05 | 99.01 | 98.34 |
| His183 Nε-Fe-His121 Nε | 82.49 | 85.75 | 81.73 | 83.61 | 83.40 |
| His119 Nε-Fe-Water 1 | 84.22 | 80.86 | 84.90 | 82.77 | 83.19 |
| His121 Nε-Fe-Water 2 | 100.53 | 96.78 | 102.02 | 101.64 | 100.24 |
| His183 Nε-Fe-Water 3 | 89.05 | 93.58 | 86.56 | 92.85 | 90.51 |

**Supplementary Table S3. List of docking results by Ginina.**

| model | affinity (kcal/mol) | intramol (kcal/mol) | CNN  pose score | CNN  affinity |
| --- | --- | --- | --- | --- |
| 1 | -4.31 | -0.23 | 0.6880 | 4.192 |
| 2 | -3.90 | -0.23 | 0.6647 | 3.959 |
| 3 | -4.03 | -0.22 | 0.6381 | 3.932 |
| 4 | -4.05 | -0.24 | 0.6347 | 3.822 |
| 5 | -3.50 | -0.44 | 0.6210 | 3.752 |
| 6 | -4.04 | -0.22 | 0.5956 | 3.845 |
| 7 | -3.44 | -0.23 | 0.5825 | 3.550 |
| 8 | -3.34 | -0.20 | 0.5806 | 3.503 |
| 9 | -3.36 | -0.21 | 0.5793 | 3.554 |
| 10 | -3.82 | -0.24 | 0.5310 | 3.742 |
| 11 | -4.03 | -0.23 | 0.5310 | 3.321 |
| 12 | -3.54 | -0.07 | 0.5185 | 3.696 |
| 13 | -3.25 | -0.24 | 0.5154 | 3.376 |
| 14 | -4.16 | -0.22 | 0.5128 | 3.456 |
| 15 | -3.57 | -0.24 | 0.4871 | 3.709 |
| 16 | -3.59 | -0.23 | 0.4850 | 3.680 |
| 17 | -3.68 | -0.23 | 0.4599 | 3.250 |
| 18 | -4.32 | -0.21 | 0.4327 | 2.953 |
| 19 | -3.64 | -0.20 | 0.4172 | 3.404 |
| 20 | -4.58 | -0.23 | 0.4155 | 2.989 |

**Supplementary Table S4.** **Structural similarity search by using the DALI server.**

| No. | PDB code | Z | rmsd | lali | nres | %id | Description |
| --- | --- | --- | --- | --- | --- | --- | --- |
| 1 | 4xxf-A | 30.2 | 1.7 | 221 | 249 | 29 | Fuculose-1-phosphate aldolase |
| 2 | 3ocr-A | 27.9 | 2.1 | 229 | 251 | 23 | Class II aldolase/adducin domain protein |
| 3 | 3ocr-B | 27.5 | 2.1 | 226 | 248 | 23 | Class II aldolase/adducin domain protein |
| 4 | 2z7b-A | 27.3 | 2.1 | 223 | 237 | 19 | 3-hydroxy-2-methylpyridine-4,5-dicarboxylate decarboxylase |
| 5 | 6btg-A | 25.0 | 2.5 | 207 | 207 | 20 | 5-deoxyribose disposal aldolase |
| 6 | 1e48-P | 24.5 | 2.6 | 203 | 206 | 18 | L-fuculose 1-phosphate aldolase |
| 7 | 6btd-A | 24.5 | 2.4 | 203 | 203 | 20 | 5-deoxyribose disposal aldolase |
| 8 | 1dzw-P | 24.4 | 2.6 | 203 | 206 | 18 | L-fuculose-1-phosphate aldolase |
| 9 | 1e4a-P | 24.3 | 2.6 | 202 | 205 | 17 | L-fuculose 1-phosphate aldolase |
| 10 | 4fua-A | 24.3 | 2.7 | 203 | 206 | 18 | L-fuculose-1-phosphate aldolase |
| 11 | 1e46-P | 24.2 | 2.6 | 203 | 206 | 18 | L-fuculose 1-phosphate aldolase |
| 12 | 1dzz-P | 24.2 | 2.5 | 202 | 208 | 18 | L-fuculose-1-phosphate aldolase |
| 13 | 1e4b-P | 24.1 | 2.5 | 200 | 206 | 19 | L-fuculose 1-phosphate aldolase |
| 14 | 1dzv-P | 24.1 | 2.5 | 200 | 206 | 18 | L-fuculose-1-phosphate aldolase |
| 15 | 1dzx-P | 24.1 | 2.5 | 202 | 208 | 18 | L-fuculose-1-phosphate aldolase |
| 16 | 2fua-A | 24.0 | 2.6 | 203 | 210 | 18 | L-fuculose-1-phosphate aldolase |
| 17 | 1e4c-P | 24.0 | 2.5 | 200 | 206 | 18 | L-fuculose 1-phosphate aldolase |
| 18 | 1e47-P | 23.9 | 2.5 | 200 | 206 | 18 | L-fuculose 1-phosphate aldolase |
| 19 | 1dzy-P | 23.9 | 2.5 | 200 | 206 | 18 | L-fuculose-1-phosphate aldolase |
| 20 | 1dzu-P | 23.9 | 2.5 | 202 | 209 | 18 | L-fuculose-1-phosphate aldolase |
| 21 | 1fua-A | 23.9 | 2.5 | 200 | 206 | 18 | L-fuculose-1-phosphate aldolase |
| 22 | 1e49-P | 23.8 | 2.5 | 200 | 206 | 18 | L-fuculose 1-phosphate aldolase |
| 23 | 7x78-A | 23.7 | 2.4 | 197 | 203 | 19 | L-fuculose phosphate aldolase |
| 24 | 4c24-A | 23.6 | 2.5 | 203 | 211 | 26 | L-fuculose phosphate aldolase |
| 25 | 4c25-A | 23.6 | 2.6 | 204 | 212 | 25 | L-fuculose phosphate aldolase |
| 26 | 6voq-A | 23.5 | 2.4 | 198 | 207 | 21 | Aldolase |
| 27 | 3fua-A | 23.5 | 2.5 | 200 | 206 | 18 | L-fuculose-1-phosphate aldolase |
| 28 | 6vop-A | 23.4 | 2.1 | 194 | 207 | 20 | Aldolase |
| 29 | 1jdi-C | 23.2 | 2.6 | 208 | 223 | 19 | L-ribulose 5 phosphate 4-epimerase |
| 30 | 1jdi-F | 23.2 | 2.6 | 208 | 223 | 19 | L-ribulose 5 phosphate 4-epimerase |
| 31 | 1jdi-E | 23.2 | 2.6 | 208 | 223 | 19 | L-ribulose 5 phosphate 4-epimerase |
| 32 | 1jdi-A | 23.1 | 2.6 | 208 | 223 | 19 | L-ribulose 5 phosphate 4-epimerase |
| 33 | 1jdi-B | 23.1 | 2.6 | 208 | 223 | 19 | L-ribulose 5 phosphate 4-epimerase |
| 34 | 1k0w-D | 23.1 | 2.6 | 209 | 223 | 19 | L-ribulose 5 phosphate 4-epimerase |
| 35 | 1jdi-D | 23.1 | 2.6 | 208 | 223 | 19 | L-ribulose 5 phosphate 4-epimerase |
| 36 | 1k0w-B | 23.0 | 2.6 | 208 | 223 | 19 | L-ribulose 5 phosphate 4-epimerase |
| 37 | 1k0w-E | 23.0 | 2.5 | 207 | 223 | 19 | L-ribulose 5 phosphate 4-epimerase |
| 38 | 1k0w-C | 23.0 | 2.6 | 209 | 223 | 19 | L-ribulose 5 phosphate 4-epimerase |
| 39 | 1k0w-A | 22.9 | 2.7 | 209 | 223 | 19 | L-ribulose 5 phosphate 4-epimerase |
| 40 | 1k0w-F | 22.8 | 2.6 | 208 | 223 | 19 | L-ribulose 5 phosphate 4-epimerase |
| 41 | 2opi-A | 21.9 | 2.7 | 198 | 203 | 15 | L-fuculose-1-phosphate aldolase |
| 42 | 2opi-B | 21.2 | 2.8 | 197 | 209 | 15 | L-fuculose-1-phosphate aldolase |
| 43 | 1pvt-A | 21.2 | 2.3 | 194 | 232 | 20 | Sugar-phosphate aldolase |
| 44 | 2fk5-A | 21.0 | 2.0 | 179 | 195 | 22 | Fuculose-1-phosphate aldolase |
| 45 | 2fk5-B | 21.0 | 2.0 | 179 | 194 | 22 | Fuculose-1-phosphate aldolase |
| 46 | 2flf-H | 20.6 | 2.0 | 178 | 187 | 22 | Fuculose-1-phosphate aldolase |
| 47 | 2flf-D | 20.5 | 2.1 | 179 | 191 | 22 | Fuculose-1-phosphate aldolase |
| 48 | 2flf-C | 20.5 | 2.0 | 177 | 191 | 22 | Fuculose-1-phosphate aldolase |
| 49 | 2flf-G | 20.5 | 2.1 | 178 | 188 | 22 | Fuculose-1-phosphate aldolase |
| 50 | 2flf-B | 20.4 | 2.1 | 179 | 191 | 22 | Fuculose-1-phosphate aldolase |

**
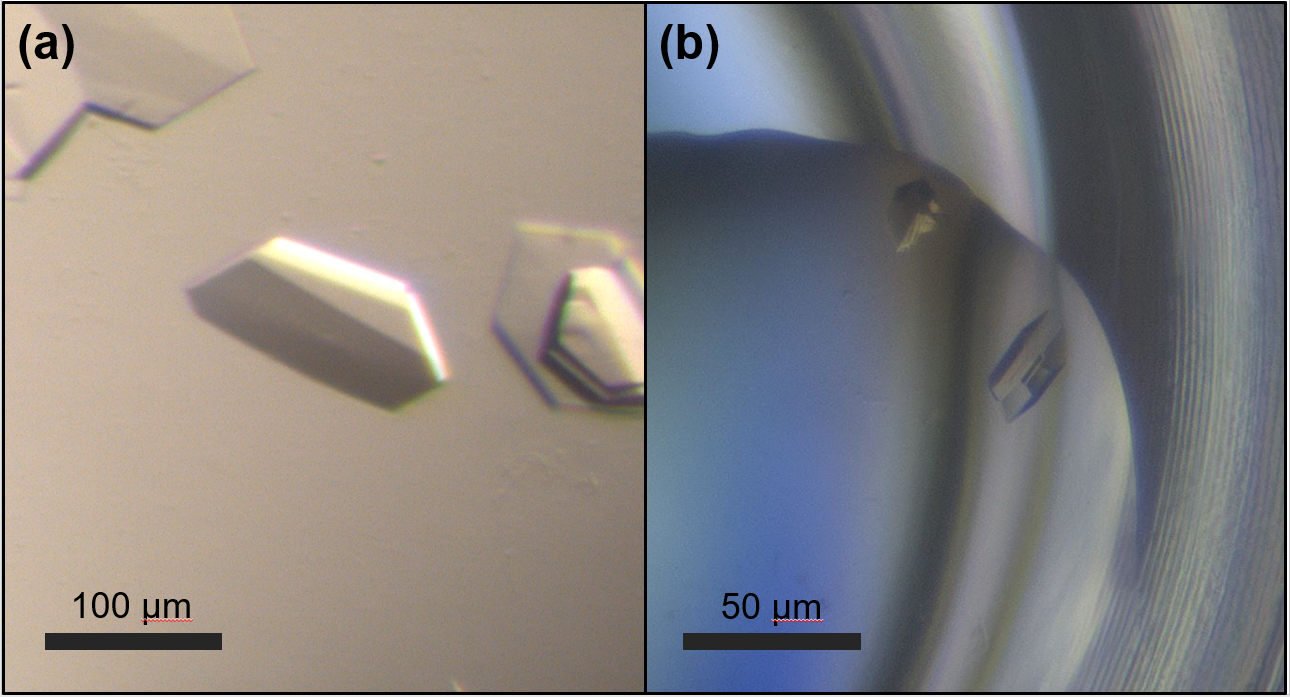
**

**Fig. S1. Crystals of (a) AfPOD and (b) AfPODdN18.**

**
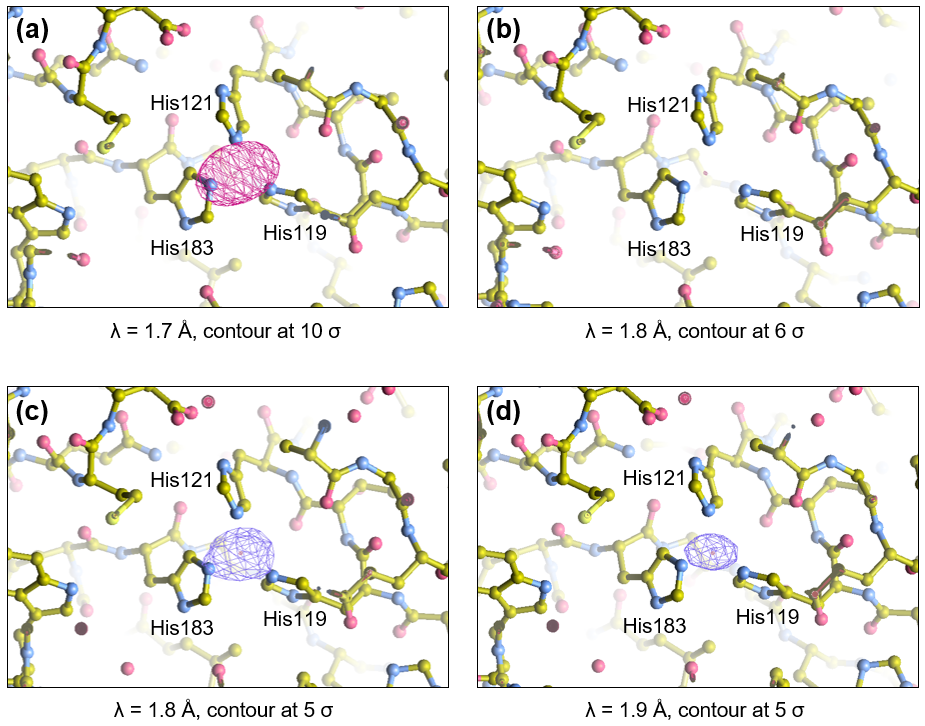
**

**Fig. S2. Identification of metal species at the active sites of AfPOD and AfPODdN18.** Anomalous difference Fourier map of AfPOD obtained by using the diffraction data collected at (**a**) 1.7 Å and (**b**) 1.8 Å. Anomalous difference Fourier map of AfPODdN18 obtained by using the diffraction data collected at (**c**) 1.8 Å and (**d**) 1.9 Å.

**
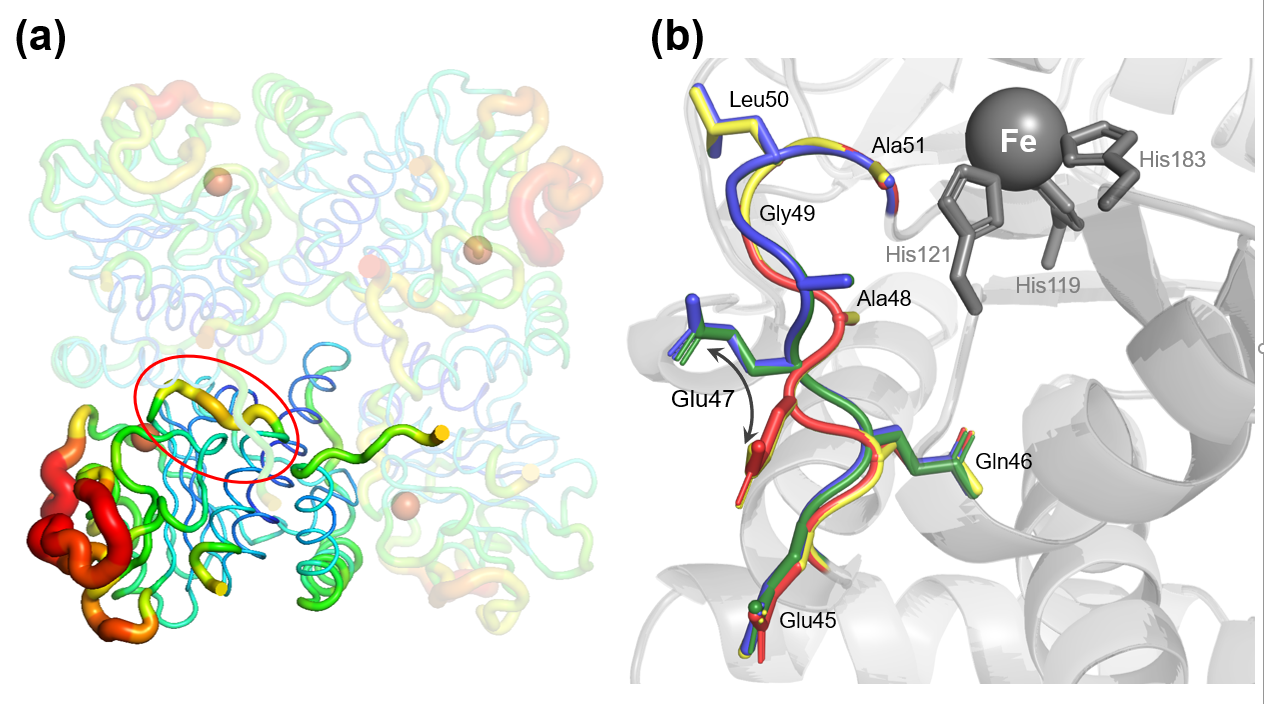
**

**Fig. S3. The mobile loop of AfPOD.** (**a**) B-factor putty representation of the AfPOD structure. An α1-β1 loop (Glu45~Ala51) near the iron center is circled in red. (**b**) The mobile loops of the AfPOD subunits A (blue), B (yellow), C (green), and D (red) have been superimposed.

**
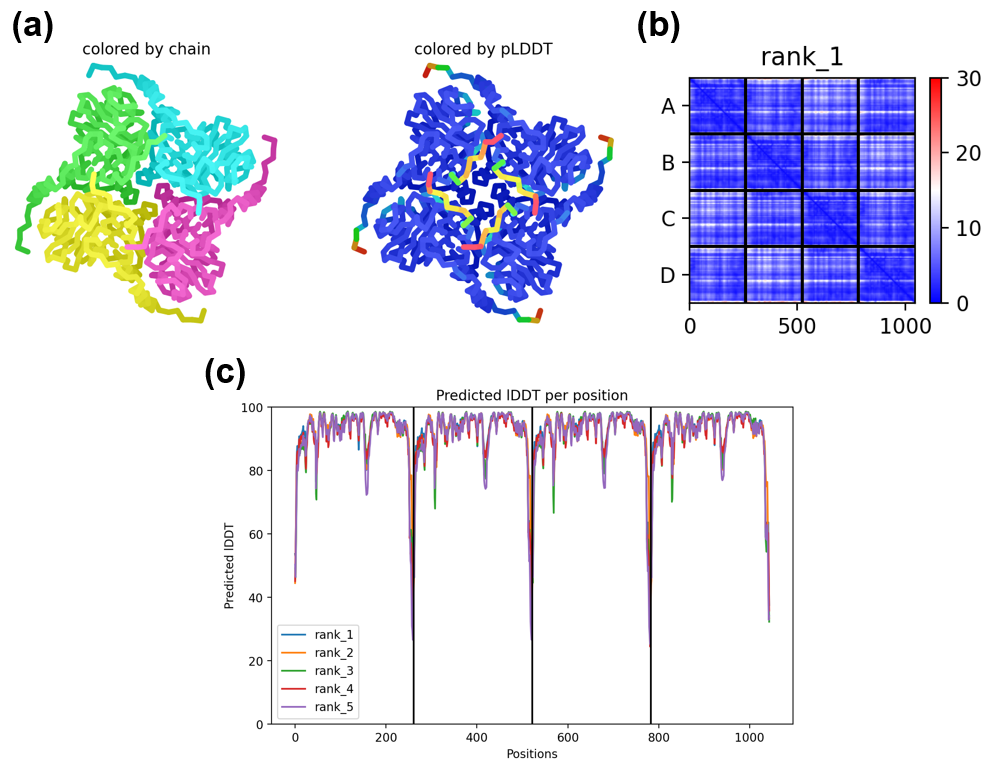
**

**Fig. S4. Confidence metrics for the predicted structure of AfPOD.** (**a**) The predicted rank_1 structural model of AfPOD homotetramer colored by chain and by pLDDT (predicted Local Distance Difference Test). (**b**) Prediction aligned error (PAE) score for the rank_1 model. This score shows the calculated error of the predicted distance for each pair of residues. Both axes indicate the position of the each amino acid. The uncertainty in the predicted distance of two amino acids is color-coded from blue (0 Å) to red (30 Å), as shown in the right bar. (**c**) The pLDDT score per position for the five models generated by AlphaFold2 for the AfPOD homotetramer. The amino acid position is plotted against the pLDDT.

**
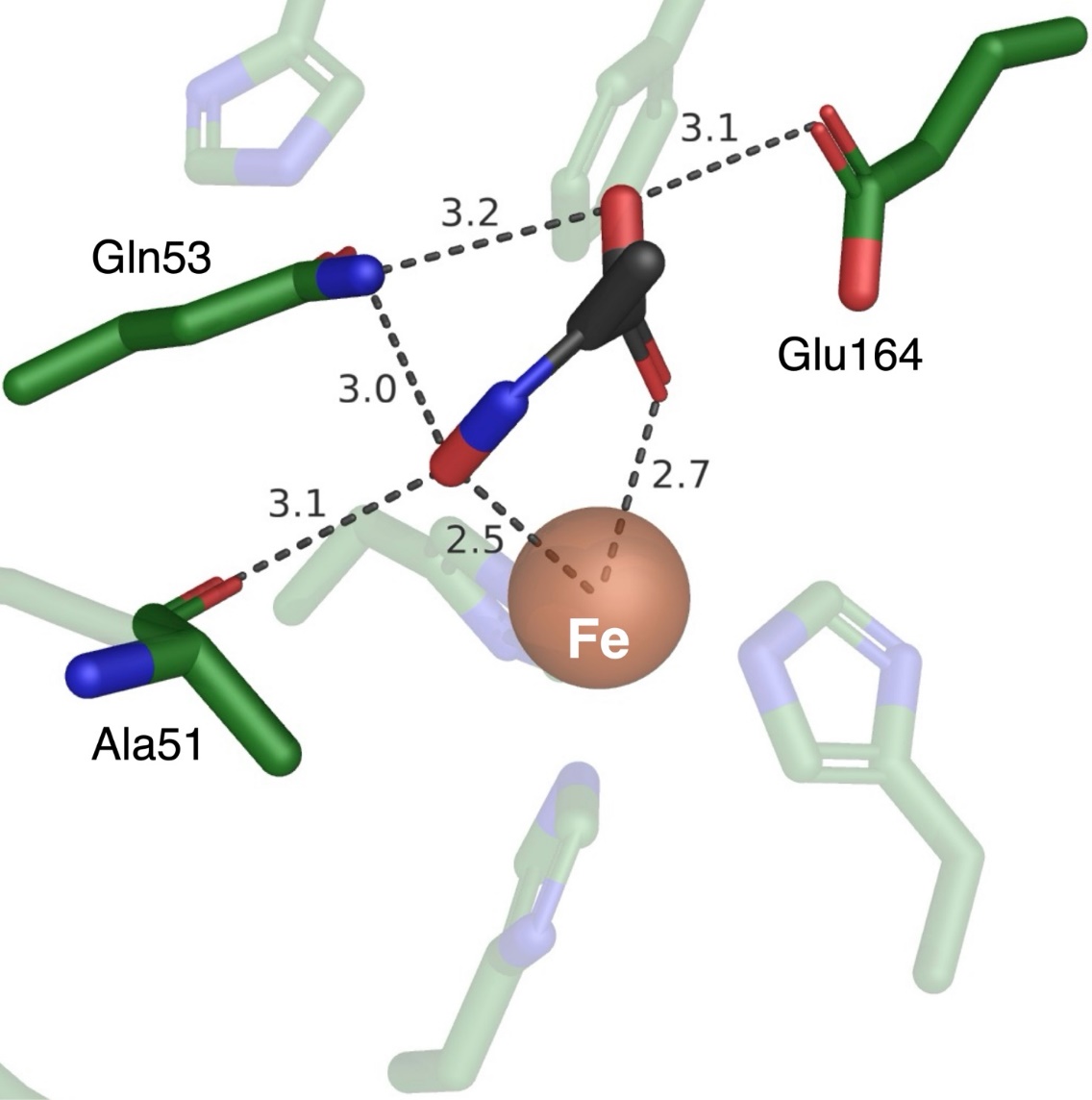
**

**Fig. S5. The docking structure with the highest pose score (model 1) of the active site of AfPOD with pyruvic oxime.**
